# Complete Mitochondrial Genomes of Three Rhinoceros Beetles (Coleoptera: Scarabaeidae: Dynastinae) and Phylogenetic Implications

**DOI:** 10.3390/biology15120953

**Published:** 2026-06-18

**Authors:** Nan Song, Renfu Shao, Qing Zhai

**Affiliations:** 1 College of Plant Protection, Henan Agricultural University, Zhengzhou 450046, China; songnan@henau.edu.cn; 2 Centre for Bioinnovation, and School of Science, Technology and Engineering, University of the Sunshine Coast, 90 Sippy Downs Drive, Sunshine Coast, QLD 4556, Australia; 3 Xizang Autonomous Region Field Scientific Observation and Research Station for Crop Pest Monitoring and Green Control, Lhasa 850000, China; 4 Institute of Vegetable, Tibet Academy of Agricultural and Animal Husbandry Sciences, Lhasa 850011, China

**Keywords:** Dynastinae, Pentodontini, Cyclocephalini, mitogenome, phylogeny

## Abstract

The subfamily Dynastinae, commonly known as rhinoceros beetles, represents one of the most morphologically striking lineages within the Scarabaeidae. This distinctiveness arises from the elaborate horns present on the head and pronotum of many species, particularly in males. Despite their significant ecological and economic importance, the phylogenetic relationships within this subfamily remain poorly understood, and available mitochondrial genomic data are remarkably scarce, hindering comprehensive phylogenomic analyses. In this study, we sequenced the complete mitochondrial genomes of three dynastine species for the first time, including the first representatives for the tribes Pentodontini and Cyclocephalini. All three genomes contain the typical set of 37 mitochondrial genes; however, a rearrangement in the tRNA gene cluster *trnQ-trnI-trnM* was observed. Given that this rearrangement is also present in other lineages within the subfamily Dynastinae but not in any other subfamilies, we propose it as a potential synapomorphy for Dynastinae. Furthermore, all three newly sequenced genomes exhibit relatively large sizes, which may be attributed to their expanded control regions. By integrating these sequences with existing Scarabaeidae mitogenome data, we reconstructed the phylogeny of Dynastinae. Our results showed that the tribes Dynastini and Oryctini were not monophyletic, suggesting the need for a taxonomic revision of these tribes. Our results also support the three-subtribe hypothesis for the tribe Dynastini.

## Simple Summary

This study advances our understanding of rhinoceros beetles (subfamily Dynastinae) by sequencing the mitochondrial genomes of three species, including the first representatives for the tribes Pentodontini and Cyclocephalini. While these genomes contain the standard set of 37 genes, we identified a tRNA gene rearrangement (*trnQ-trnI-trnM*) that is likely a shared derived trait (synapomorphy) for the subfamily. Additionally, the study reveals that these genomes contain expanded, complex non-coding control regions, which contribute to their larger overall size. By integrating these data into a broader phylogenetic analysis, our results showed that the tribes Dynastini and Oryctini were not monophyletic, suggesting the need for a taxonomic revision of these tribes. Notably, our findings corroborate the three-subtribe hypothesis within the tribe Dynastini, providing a more robust framework for future evolutionary research on these iconic beetles.

## 1. Introduction

The family Scarabaeidae (Coleoptera) is among the most diverse beetle groups, comprising more than 35,000 described species [1]. Within this massive radiation, the subfamily Dynastinae, commonly known as rhinoceros beetles, stands out as one of the most morphologically striking lineages. This common name is derived from the presence of elaborate horns on the head and/or pronotum of many species, particularly the males. These structures have rendered rhinoceros beetles both iconic subjects for insect collectors and primary models for evolutionary biologists investigating sexual selection and the development of secondary sexual characters [2]. Research into these structures has elucidated the evolutionary and developmental mechanisms driving the diversification of such extreme phenotypes. These horns, often utilized as weapons in male–male competition to secure access to mates and breeding sites, are products of intense sexual selection [3,4,5]. Furthermore, many dynastine species exhibit bimodal allometry, characterized by the development of dramatically different horn phenotypes between large and small males [6,7].

With approximately 225 genera and 1500 species, Dynastinae are distributed worldwide with their highest taxonomic and morphological diversity concentrated in the tropics [8]. As a primarily phytophagous scarab lineage, these beetles occupy distinct ecological niches across life stages: larvae typically feed on roots or decompose organic matter, while adults generally consume fruits, plant exudates, or decaying vegetation [9]. The subfamily Dynastinae comprises eight tribes: Agaocephalini, Cyclocephalini, Dynastini, Hexodontini, Oryctini, Oryctoderini, Pentodontini and Phileurini [10]. Among these, the tribe Cyclocephalini comprises approximately 500 species across 15 genera and includes essential pollinators of aroids and palms, as well as several significant agricultural pests and invasive species. Pentodontini stands as the largest tribe within the subfamily, containing roughly 100 genera and 550 species. Additionally, the tribe Dynastini, the giant rhinoceros beetle, contains some of the world’s largest insects and exhibits extreme sexual dimorphism [2]. Given their ecological and economic importance, a robust phylogenetic framework is essential [11]. Such a framework would provide a critical foundation for predicting species invasiveness in novel environments, elucidating co-evolutionary dynamics with host plants, and tracing the evolutionary origins of their remarkable traits [12]. Nevertheless, comprehensive phylogenetic studies of Dynastinae remain strikingly limited.

Mitogenome sequences have been utilized extensively to investigate beetle relationships across various taxonomic scales [13,14,15,16,17,18,19,20,21,22,23,24]. In particular, the advent of next-generation sequencing technologies has facilitated the efficient, simultaneous acquisition of complete mitogenomes for large-scale insect sampling. Despite these advancements, genomic data for the subfamily Dynastinae remains notably sparse. As of January 2026, the mitogenomes of only 31 rhinoceros beetle species and subspecies are available in GenBank, and few published studies have leveraged mitogenomic data to reconstruct the group’s phylogeny. This lack of comprehensive data continues to impede molecular research into the phylogenetic relationships of this subfamily.

In this study, we sequenced the mitogenomes of three rhinoceros beetle species, *Cyclocephala signaticollis*, *Dasygnathus* sp., and *Xylotrupes australicus*, representing three distinct genera, two of which were sequenced here for the first time. These newly generated data were integrated with publicly available Scarabaeidae mitogenomes to infer a preliminary phylogeny of Dynastinae. This study aims to provide new insights into the evolutionary history and diversification of this subfamily.

## 2. Materials and Methods

### 2.1. Specimens Collection

Individual insect specimens were hand-collected at the University of the Sunshine Coast (UniSC, Sippy Downs; 26°42′58″ S, 153°03′34″ E) between November and December 2023. No specific permits were required for the insect sampling conducted in this study. The specimens were preserved in absolute ethanol at −20 °C until DNA extraction. Species identification was performed using an integrated approach combining adult morphological characteristics and molecular analysis. For morphological identification, all specimens were examined under a Leica M205 A stereomicroscope (Leica Microsystems, Wetzlar, Germany) using diagnostic keys and descriptions in taxonomic literature [2,25,26,27,28,29]. The main diagnostic characters included a pale testaceous dorsum with distinct, symmetrical dark pronotal and cephalic markings for *Cyclocephala signaticollis*; a large, dark chocolate-brown to black body with striking sexual dimorphism—specifically featuring a small, hornless, coarsely punctured matte pronotum in females—for *Xylotrupes australicus*; and a pitch-black body with golden ventral setae, paired with dense, brush-like maxillary and labial setae for *Dasygnathus* sp., whose females had a reduced cephalic ridge. For molecular identification, mitochondrial *cox1* gene fragments were sequenced and compared against public databases, specifically the Barcode of Life Data System (BOLD: https://id.boldsystems.org/, accessed on 18 June 2026) [30]. Species names were only confirmed if the sequence match yielded an identity percentage (ID%) greater than 99%.

### 2.2. DNA Extraction

Genomic DNA was extracted from the leg muscle tissue of a single specimen preserved in 100% ethanol. Extraction was performed using the TIANamp Genomic DNA Kit (Tiangen Biotech Co., Ltd., Beijing, China), strictly adhering to the manufacturer’s protocol. Following extraction, the DNA concentration of each sample was quantified using a Q5000 nucleic acid protein analyzer (Quawell Technology, Inc., San Jose, CA, USA).

### 2.3. Genome Sequencing and Quality Control

Total genomic sequencing was performed by Personal Biotechnology Co., Ltd. (Shanghai, China). Genomic sequencing was performed using the DNBSEQ-T7RS platform. Prior to sequencing, genomic DNA libraries were constructed with a target insert size of 400 bp using the TruSeq Nano DNA High Throughput Library Prep Kit in accordance with the manufacturer’s protocol. High-throughput sequencing was subsequently conducted using the DNBSEQ-T7RS High-throughput Sequencing Set V3.0, using a paired-end 150 bp strategy. The raw data underwent rigorous quality control, yielding 27 Gbp for *C. signaticollis*, 27.9 Gbp for *Dasygnathus* sp., and 26.7 Gbp for *X. australicus*. Fastp v0.23 [31] was used for initial assessment, and Trimmomatic v0.32 [32] was employed to remove adapter sequences and filter low-quality reads. Only high-quality reads with a Q30 score > 95% were retained for subsequent de novo assembly. Genome sequencing statistics are summarized in Table S1.

### 2.4. Mitogenome Assembly and Annotation

The complete mitogenome was assembled from the filtered paired-end reads using GetOrganelle v1.7.7.1 [33]. The assembly parameters were set to “animal mitochondrial” (animal_mt) using GetOrganelleDB 0.0.1 as the reference database. The circularity and completeness of the assembled mitogenomes were evaluated using the automated, graph-based validation workflow in GetOrganelle v1.7.7.1 [33]. Completeness was achieved through GetOrganelle’s seed-led recruitment strategy, which used a multi-K-mer gradient (ranging from 21 to 85) to iteratively extend mitochondrial genome reads until data saturation. Circularity was verified algorithmically when the software resolved terminal repeats within the assembly graph (FASTG/GFA format) into a single, closed-loop path, exporting the sequence with a “circular” designation. Assembly integrity was further validated downstream by confirming the presence of all 37 typical metazoan mitochondrial genes. Mapping to the mitogenome assembly was performed using Geneious R11 [34], which was also used to generate nucleotide coverage statistics.

Preliminary de novo annotation of the mitogenome was conducted using the MITOS2 [35]. To ensure accuracy, the boundaries of protein-coding genes and the two rRNA genes were manually refined through multiple sequence alignments with mitogenomes of closely related species available in GenBank. The secondary structures of tRNAs were initially predicted using MITOS v2 and subsequently confirmed with tRNAscan-SE v2.0 (https://trna.ucsc.edu/tRNAscan-SE/, accessed on 18 June 2026) [36]. The tRNAscan-SE v2.0 analysis utilized the ‘Invertebrate Mito’ genetic code for isotype prediction and a score cutoff of 10.0. The newly sequenced mitochondrial genomes have been submitted to the NCBI GenBank database; their respective accession numbers are as follows: PZ322948-PZ322950.

PhyloSuite v2 [37,38] was utilized for downstream analyses, including gene extraction, mitochondrial genome statistics calculation, as well as the generation of Relative Synonymous Codon Usage (RSCU) values and gene order visualization.

### 2.5. Sequence Alignment and Dataset Construction

Multiple sequence alignments of the 13 protein-coding genes (PCGs) were generated using MACSE v2 [39], which accounts for the invertebrate mitochondrial genetic code and prevents disruptions to the open reading frame. These alignments were automatically trimmed using the built-in refinement tools in MACSE v2. For the ribosomal RNA genes (rRNAs), sequences were aligned using the MAFFT v7 [40] with the E-INS-i strategy, followed by automated trimming with trimAl v1.2 [41] to remove poorly aligned regions.

All individual gene alignments were concatenated using FASconCAT-G v1.0 [42]. To evaluate the phylogenetic signal and potential biases, three distinct datasets were compiled: (1) PCG_rRNA: Nucleotide sequences of the 13 PCGs and 2 rRNAs; (2) PCG_nt: Nucleotide sequences of the 13 PCGs; (3) PCG_AA: Amino acid sequences of the 13 PCGs.

### 2.6. Phylogenetic Analyses

The species included in the phylogenetic analysis are listed in Table 1. The dataset comprises 38 species representing five tribes of the subfamily Dynastinae as the ingroup, with three species from Cetoniinae and three from Rutelinae serving as outgroups. Phylogenetic reconstruction was performed using Maximum Likelihood (ML) and Bayesian Inference (BI) criteria. For the ML analyses, we used IQ-TREE v2.0 [43]. The datasets were partitioned by gene, and the optimal substitution models and partitioning schemes were determined using ModelFinder [44]. Branch support was assessed with 10,000 ultrafast bootstrap (BS) [45] replicates ensure the robustness of the topology.

BI analyses were conducted using PhyloBayes MPI v1.8 [46]. To account for site-specific substitution processes, the site-heterogeneous CAT-GTR model was applied to the nucleotide datasets, while the CAT-mtArt model was used for the amino acid dataset. Two independent chains were run in parallel starting from random topologies [47]. Convergence was monitored using the bpcomp and tracecomp programs. The analysis was considered to have reached stationarity when the maximum discrepancy (maxdiff) was <0.1 and the minimum effective size was >300, after discarding the initial 1000 cycles as burn-in. The final consensus tree and posterior probabilities (BPP) were generated from the remaining trees.

## 3. Results

### 3.1. Mitogenome Organization and Composition

The three newly sequenced mitogenomes range in length from 17,274 bp (*Dasygnathus* sp.) to 19,258 bp (*Cyclocephala signaticollis*). GetOrganelle exported the assembled sequence of *C. signaticollis* with a “circular” designation. Although the assemblies of the other two species were not explicitly flagged as circular by GetOrganelle, subsequent annotation successfully identified the complete set of 37 typical mitochondrial genes in both species. While the three newly sequenced mitogenomes are larger than that of *Drosophila yakuba* (16,019 bp), they remain within the typical insect mitogenome range of 15–18 kb [48]. The observed size expansion is primarily attributed to large non-coding regions situated between *rrnS* and *trnQ*, measuring 4,390 bp in *C. signaticollis*, 2511 bp in *Dasygnathus* sp., and 2863 bp in *Xylotrupes australicus*. The mean base coverage was 32,277.3× for *C. signaticollis* (range: 3–58,549), 8353.8× for *Dasygnathus* sp. (range: 2–42,882), and 19,713.9× for *X. australicus* (range: 24–44,344).

**Figure 1:**
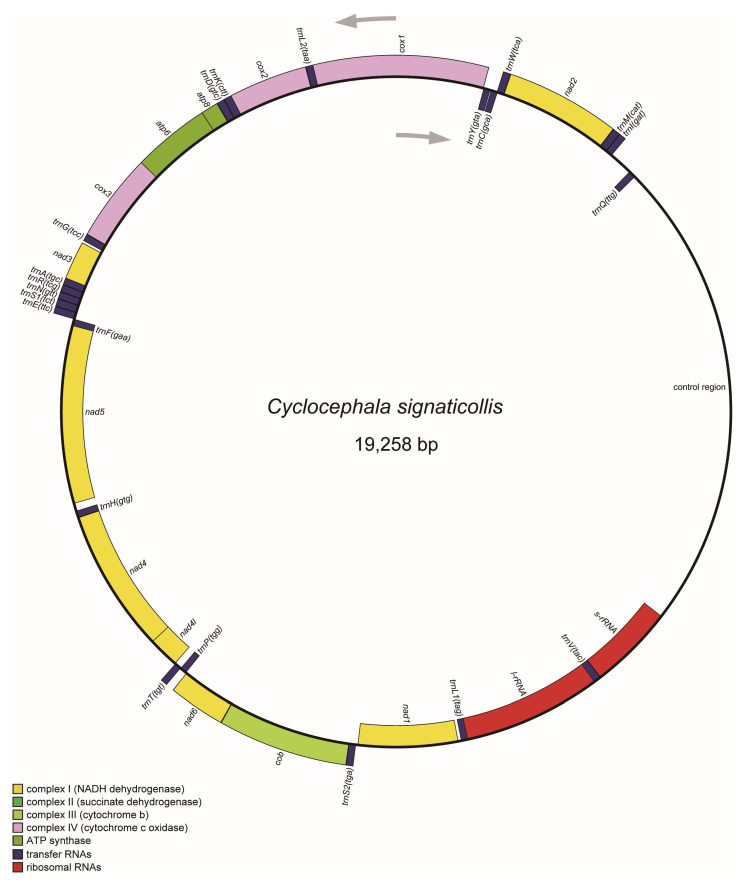
Circular map of the *Cyclocephala signaticollis* mitochondrial genome. Genes are abbreviated according to MITOS nomenclature. Arrows indicate the transcriptional orientation of the strands.

Each mitogenome contains the standard set of 37 genes: 13 protein-coding genes (PCGs), 22 tRNA genes, and 2 rRNA genes (Figure 1, Supplementary Figure S1, and Table S2). These genomes exhibit a heavy A+T bias, with content ranging from 75.3% (*Dasygnathus* sp.) to 76.7% (*X. australicus*). Additionally, the major strand shows a positive AT skew, varying between 0.02 (*Dasygnathus* sp.) and 0.054 (*C. signaticollis*).

### 3.2. Protein-Coding Genes

The total length of the 13 protein-coding genes (PCGs) across the newly sequenced mitogenomes ranged from 11,114 bp (*X. australicus*) to 11,151 bp (*Dasygnathus* sp.), with an average A+T content between 74% (*C. signaticollis*) and 76% (*X. australicus*). All PCGs utilized the typical start codon ATN (including ATG, ATT, and ATA). Most PCGs across all species terminated with conventional stop codons (TAA or TAG). However, incomplete stop codons (TA or T) were identified in *cox1-3* and *nad5* for three species, as well as in *nad3* for *C. signaticollis*.

**Figure 2:**
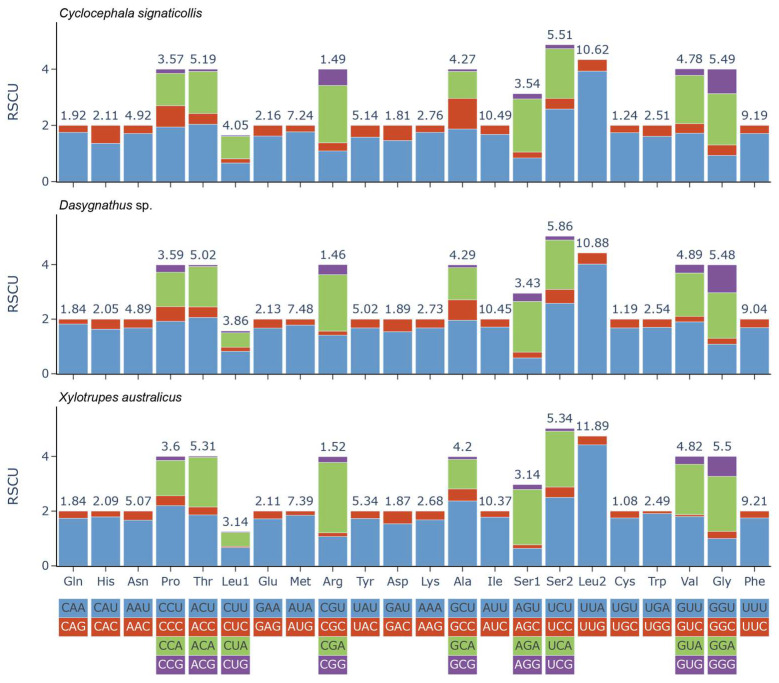
Relative synonymous codon usage (RSCU) in protein-coding genes. Data are derived from the three newly sequenced mitogenomes.

Relative synonymous codon usage (RSCU) is consistent across all three species, with UUA (Leu), UCU (Ser), CGU (Arg), and GGA (Gly) identified as the most frequently used codons (Figure 2). All dominant codons are A+T rich, suggesting that a strong AT mutation bias significantly influences codon selection.

### 3.3. Transfer RNA Genes and Ribosomal RNA Genes

**Figure 3:**
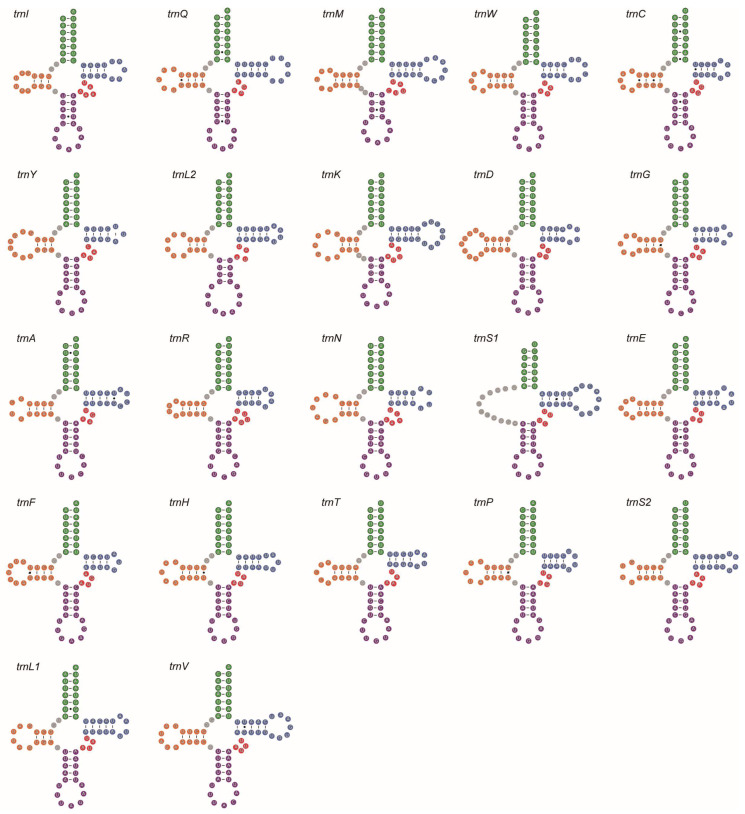
Predicted secondary structures of the 22 mitochondrial tRNAs in *Cyclocephala signaticollis*. Functional arms are color-coded: green (amino-acyl arm), orange (dihydrouracil arm), purple (anticodon arm), red (variable arm), and blue (TψC arm). Watson–Crick pairings are indicated by bars; G-U wobble pairs are indicated by dots.

A total of 22 tRNA genes were identified in the mitogenomes of each of the three newly sequenced species. The lengths of these tRNA genes were largely consistent across species, with most specific tRNA types exhibiting identical lengths. These sequences ranged from 62 bp (found in *trnC* of *Dasygnathus* sp.) to 71 bp (found in *trnK* across all three species). The tRNA secondary structures for *C. signaticollis* are illustrated in Figure 3. All tRNA genes have the canonical cloverleaf structure, except for *trnS*_1_, which lacks the DHU arm. The other two mitogenomes exhibit highly similar tRNA gene sequences and secondary structures.

The two rRNA genes, *rrnS* and *rrnL*, are both encoded on the minor strand. *rrnS* is located between *trnV* and the control region, ranging from 792 to 796 bp in length. *rrnL* is located between *trnL_1_* and *trnV*, varying in length from 1287 bp to 1325 bp.

### 3.4. Gene Rearrangements

Compared to the putative ancestral insect mitogenome, a distinct gene rearrangement was observed within the sampled taxa. While the six outgroup mitogenomes retain the ancestral tRNA gene order *trnI-trnQ-trnM*, all three newly sequenced mitogenomes and those of analyzed Dynastinae species, exhibit a derived gene order *trnQ-trnI-trnM* (Figure S1). This conserved rearrangement across the subfamily suggests a shared evolutionary event within the Dynastinae lineage that deviates from the ancestral insect mitochondrial gene arrangement.

### 3.5. Non-Coding Regions of the New Mitogenomes

The mitogenomes of the three Dynastinae species, *C. signaticollis*, *Dasygnathus* sp., and *X. australicus*, consistently feature two sections of the control region (i.e., CR1 and CR2 in Figure S2), characterized by high A+T content and complex repetitive architectures. This dual-section configuration indicates an ancestral duplication event within the lineage, with each section evolving specialized structural and functional roles.

In *C. signaticollis*, CR1 (2009 bp) is dominated by a massive tandem repeat array consisting of 7 copies of a 185-bp macro-repeat. The near-perfect sequence conservation among these units suggests recent expansion maintained by concerted evolution. Conversely, CR2 (2380 bp) exhibits a more complex, non-periodic architecture enriched with short microsatellites and putative regulatory signals, including long poly-T stretches and hairpin-forming sequences. While CR1 appears to drive genomic length variation through copy number fluctuations, CR2 likely hosts the primary replication and transcriptional control signals.

*Dasygnathus* sp. exhibits highly asymmetrical structural complexity between its two non-coding regions. CR1 (174 bp) is a truncated, non-repetitive segment primarily composed of simple (*TA*)*_n_* microsatellites. In contrast, CR2 (2336 bp) serves as the functional centerpiece for regulation and length variation. Its upstream region contains a tandem repeat array of three ~120 bp units (core motif: ATAAATCTCCCTG), while the downstream regulatory domain harbors extended poly-T/A tracks and potential stem-loop structures associated with the origin of light-strand replication. The 3′ terminus is further distinguished by unique G-clusters (e.g., GGGGG), which may facilitate specialized protein-binding or transcriptional termination.

For *X. australicus*, CR1 (2445 bp) contains central (*TA*)*_n_* or (*AT*)*_n_* motifs followed by a 3′ terminal region defined by three copies of a 91-bp macro-repeat, a hallmark of the Dynastinae subfamily. CR2 (419 bp), though significantly shorter, serves as a localized source of length polymorphism via two nearly identical (~98% identity) 123 bp tandem repeats. With an A+T content of 82.3%, CR2 is enriched with poly-A/T tracks indicative of replication slippage. Its 5′ flanking region (1–170 bp) contains potentially critical functional motifs, including a poly-T stretch and an A-rich promoter or protein-binding site (‘AAAAAACTCCATAAA’).

### 3.6. Phylogenetic Reconstruction

**Figure 4:**
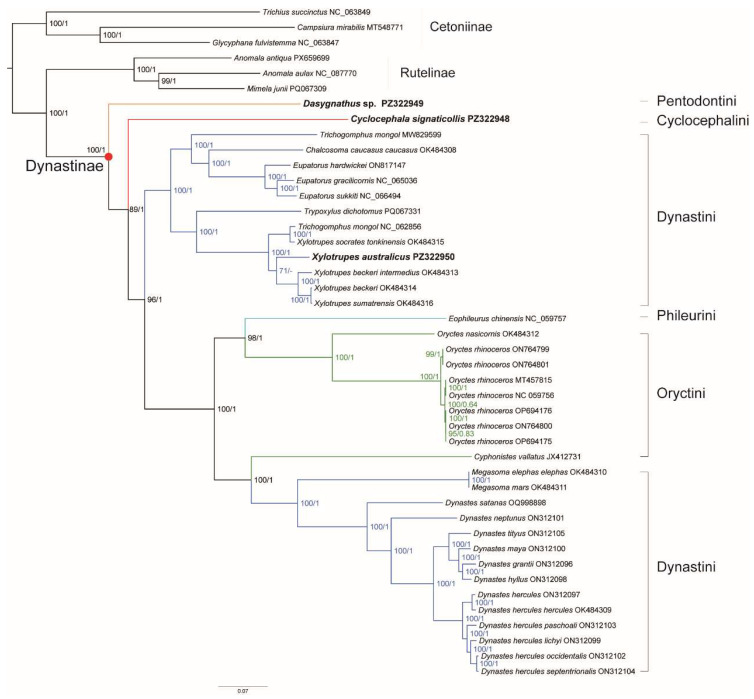
Phylogenetic relationships of Dynastinae inferred via Maximum Likelihood analysis. The tree is based on the PCGrRNA dataset. The Bayesian tree based on the same dataset had a topology highly similar to that of the ML tree. Bootstrap support values and posterior probabilities are indicated at the nodes.

ML and BI analyses across all datasets yielded highly similar tree topologies (Figure 4 and Figures S3–S7). Within the subfamily Dynastinae, the newly sequenced *Dasygnathus* sp. (representing Pentodontini) was recovered as the sister species to all remaining taxa. *C. signaticollis* (representing Cyclocephalini), also newly sequenced, formed the subsequent diverging lineage. Both Dynastini and Oryctini were recovered as non-monophyletic. Specifically, Dynastini was rendered polyphyletic by the nested placements of Phileurini and Oryctini. For the species of Dynastini, two distinct lineages were identified: one comprising *Trichogomphus*, *Chalcosoma*, *Eupatorus*, *Trypoxylus*, and *Xylotrupes*, and another consisting of *Megasoma* and *Dynastes*. Similarly, Oryctini was found to be non-monophyletic due to the disparate phylogenetic positions of *Oryctes* and *Cyphonistes*.

At the generic level, all genera represented by more than two species were confirmed as monophyletic. However, the phylogenetic placement of *Trichogomphus mongol* (retrieved from GenBank) raised concerns regarding its taxonomic identity. Two *T. mongol* mitogenomes from NCBI are separated: NC_062856 nested within the genus *Xylotrupes*, whereas MW829599 sister to a clade comprising *Chalcosoma* and *Eupatorus*. A BOLD system identification query revealed that the *cox1* sequence of NC_062856 shared 100% identity with *T. mongol* but also 98.54% identity with *Xylotrupes socrates*. Conversely, the *cox1* sequence of MW829599 matched only *T. mongol* with no high-identity matches to *Xylotrupes*. Given its phylogenetic position and high sequence similarity, we propose that the GenBank entry for *T. mongol* (NC_062856) may be a misidentification and likely represents *X. socrates*.

## 4. Discussion

### 4.1. Mitochondrial Gene Rearrangements and Phylogenetic Utility

The utility of mitochondrial gene rearrangements as phylogenetic markers has been investigated for some time, with several studies validating their viability as informative characters. In insect phylogenetics, numerous studies have attempted to correlate these rearrangements with taxonomic divergence. While mitochondrial gene rearrangements are notably frequent and diverse in Hymenoptera, recent findings suggest no significant correlation between these genomic events and taxonomic divergence within a robust phylogenetic framework [49]. Our results support the specific tRNA gene rearrangement *trnQ-trnI-trnM* as a synapomorphy for the subfamily Dynastinae [13]. This observation is consistent with a previous mitogenomic study by He et al. [17]. Although the sampling of He et al. was relatively limited and focused exclusively on the genus *Dynastes*, their findings similarly suggested this rearrangement as a diagnostic feature of the subfamily Dynastinae. The emergence of such rearrangements may be linked to the unique life histories and ecological traits of Dynastinae. In Dynastinae, these traits are characterized by intense sexual selection and localized male–male competition for resources (e.g., specific mating and breeding sites). This ecological pressure has driven the evolution of extreme secondary sexual characters, such as elaborate cephalic or pronotal horns, as well as complex developmental strategies like bimodal allometry between large and small males [3,4,5,6,7].

### 4.2. Phylogenetic Relationships Within Dynastinae

Current phylogenetic study on Dynastinae remains remarkably limited, leaving the monophyly of several tribes in doubt. Although broad-scale analyses of Coleoptera or Scarabaeoidea have included rhinoceros beetles, taxon sampling has been insufficient to resolve internal relationships [50,51]. For instance, the phylogenomic analysis by Dietz et al. [52] included only two dynastine species; while their results recovered Dynastinae and Rutelinae as sister groups (together forming a sister clade to Cetoniinae), the tribal relationships within Dynastinae remained poorly understood [2].

In the present study, we included five of the eight recognized dynastine tribes (Pentodontini, Cyclocephalini, Dynastini, Phileurini, and Oryctini) in our phylogenetic analysis, featuring three newly sequenced mitogenomes alongside 41 mitogenomes retrieved from the NCBI GenBank database (Table 1). Although our overall sampling remains limited, the addition of newly sequenced mitogenomes—particularly for the tribes Pentodontini and Cyclocephalini and the genus *Xylotrupes*—provides critical new data. These expanded resources offer fresh insights into the evolutionary history of the subfamily.

Despite the significant importance of certain Cyclocephalini species as agricultural pests or invasive threats, the tribe’s phylogeny and internal classification have not been rigorously examined [53]. Previous morphological analysis of 77 adult characters previously suggested that Cyclocephalini, as currently circumscribed, is non-monophyletic, and that the genus *Cyclocephala* itself requires revision [12]. Historically, Endrödi [54,55] considered Cyclocephalini the most primitive dynastine tribe due to the characters shared with Rutelinae; however, Endrödi’s methodology for polarizing characters into primitive and derived states has been widely questioned. Our mitogenomic analysis recovered Cyclocephalini as the second diverging clade within Dynastinae, after the divergence of Pentodontini.

While Endrödi [55] hypothesized a close relationship between Pentodontini and Oryctini, our results did not support this relationship. Instead, we found Oryctini to be non-monophyletic and nested within a larger clade. Specifically, *Oryctes* was sister to Phileurini, while *Cyphonistes* was sister to a clade comprising *Megasoma* and *Dynastes*. Our results were consistent with Gunter et al. [50], whose analysis of 22 species also failed to support the monophyly of Pentodontini and Phileurini.

Previous research by Rowland and Miller [56] divided the tribe into three subtribes: Dynastina, Xylotrupina, and Chalcosomina, with the latter two forming a large “Asian/Oceanian clade”. Our findings are consistent with this subtribal classification, recovering three distinct clades: *Chalcosoma* + *Eupatorus* (Chalcosomina), *Trypoxylus* + *Xylotrupes* (Xylotrupina), and *Megasoma* + *Dynastes* (Dynastina). Furthermore, the sister-group relationship between Chalcosomina and Xylotrupina is consistent with the arrangement proposed by Rowland and Miller [56] and Jin et al. [57].

A notable representative in our study is the newly sequenced *Xylotrupes australicus*, an iconic large insect and a prominent representative of Dynastini in Australia, due to its remarkable size, distinct morphological traits, and high public and scientific visibility [58]. While Jin et al. [57] recovered a monophyletic Dynastini using a combination of mitochondrial (*cox2*, *rrnL*) and nuclear (*H3*, *ArgKin*) genes, our analyses consistently recovered the tribe as non-monophyletic. This discrepancy is likely due to differences in taxon sampling. Jin et al. [57] did not include representatives of Phileurini and Oryctini, which were interspersed within the Dynastini lineage in our analysis (Figure 4).

At the generic level, our analysis supported the monophyly of all genera represented by two or more species in the current study (Figure 4). In particular, we confirmed the monophyly of *Xylotrupes* and its sister-group relationship with *Trypoxylus dichotomus*, a finding that corroborates previous morphological analyses [59] and reinforces the stability of these relationships within the mitogenomic framework.

## 5. Conclusions

In this study, we sequenced the complete mitogenomes of three rhinoceros beetle species, including the first representatives for the tribes Pentodontini and Cyclocephalini. This expansion of mitogenomic data significantly contributes to our understanding of Dynastinae at the tribal level. Our analyses supported the hypothesis that a specific tRNA gene rearrangement (*trnQ-trnI-trnM*) serves as a synapomorphy for the subfamily, providing molecular support for its monophyly. Furthermore, our mitogenomic phylogenetic reconstruction demonstrates that the tribes Dynastini and Oryctini are not monophyletic as currently circumscribed. Notably, we recovered Pentodontini and Cyclocephalini as relatively basal clades within the Dynastinae, providing new insights into the subfamily’s early evolutionary divergence. Within the tribe Dynastini, our results corroborate the three-subtribe hypothesis proposed by Rowland and Miller [56]. These findings establish a vital foundation for future large-scale phylogenetic analyses and provide a refined framework for understanding the evolutionary diversification of rhinoceros beetles.

## Figures and Tables

**Table 1:** Taxonomic information and mitogenome lengths for the species included in this study.

Accession Number	Organism	Subfamily	Tribe	Full Length (bp)
PZ322948	***Cyclocephala signaticollis***	Dynastinae	Cyclocephalini	19,258
OK484308	*Chalcosoma caucasus caucasus*	Dynastinae	Dynastini	19,444
ON312096	*Dynastes grantii*	Dynastinae	Dynastini	25,060
OK484309	*Dynastes hercules hercules*	Dynastinae	Dynastini	17,813
ON312099	*Dynastes hercules lichyi*	Dynastinae	Dynastini	24,357
ON312102	*Dynastes hercules occidentalis*	Dynastinae	Dynastini	24,546
ON312103	*Dynastes hercules paschoali*	Dynastinae	Dynastini	25,593
ON312104	*Dynastes hercules septentrionalis*	Dynastinae	Dynastini	24,784
ON312097	*Dynastes hercules*	Dynastinae	Dynastini	25,542
ON312098	*Dynastes hyllus*	Dynastinae	Dynastini	24,190
ON312100	*Dynastes maya*	Dynastinae	Dynastini	27,085
ON312101	*Dynastes neptunus*	Dynastinae	Dynastini	23695
OQ998898	*Dynastes satanas*	Dynastinae	Dynastini	16,973
ON312105	*Dynastes tityus*	Dynastinae	Dynastini	28,021
NC_065036	*Eupatorus gracilicornis*	Dynastinae	Dynastini	18,391
ON817147	*Eupatorus hardwickei*	Dynastinae	Dynastini	18,494
NC_066494	*Eupatorus sukkiti*	Dynastinae	Dynastini	18,445
OK484310	*Megasoma elephas elephas*	Dynastinae	Dynastini	16,785
OK484311	*Megasoma mars*	Dynastinae	Dynastini	16,983
PQ067331	*Trypoxylus dichotomus*	Dynastinae	Dynastini	19,189
PZ322950	***Xylotrupes australicus***	Dynastinae	Dynastini	17,573
OK484314	*Xylotrupes beckeri*	Dynastinae	Dynastini	18,434
OK484313	*Xylotrupes beckeri intermedius*	Dynastinae	Dynastini	18,567
OK484315	*Xylotrupes socrates tonkinensis*	Dynastinae	Dynastini	18,660
OK484316	*Xylotrupes sumatrensis*	Dynastinae	Dynastini	19,687
JX412731	*Cyphonistes vallatus*	Dynastinae	Oryctini	11,629
OK484312	*Oryctes nasicornis*	Dynastinae	Oryctini	20,396
MT457815	*Oryctes rhinoceros*	Dynastinae	Oryctini	20,898
NC_059756	*Oryctes rhinoceros*	Dynastinae	Oryctini	15,339
ON764799	*Oryctes rhinoceros*	Dynastinae	Oryctini	15,315
ON764800	*Oryctes rhinoceros*	Dynastinae	Oryctini	15,475
ON764801	*Oryctes rhinoceros*	Dynastinae	Oryctini	17,275
OP694175	*Oryctes rhinoceros*	Dynastinae	Oryctini	15,484
OP694176	*Oryctes rhinoceros*	Dynastinae	Oryctini	17,142
NC_062856	*Trichogomphus mongol*	Dynastinae	Oryctini	17,377
MW829599	*Trichogomphus mongol*	Dynastinae	Oryctini	16,737
PZ322949	***Dasygnathus* sp.**	Dynastinae	Pentodontini	17,274
NC_059757	*Eophileurus chinensis*	Dynastinae	Phileurini	16,624
NC_063847	*Glycyphana fulvistemma*	Cetoniinae	Cetoniini	16,701
MT548771	*Campsiura mirabilis*	Cetoniinae	Cremastocheilini	16,123
NC_063849	*Trichius succinctus*	Cetoniinae	Trichiini	18,358
PX659699	*Anomala antiqua*	Rutelinae	Anomalini	16,430
NC_087770	*Anomala aulax*	Rutelinae	Anomalini	16,246
PQ067309	*Mimela junii*	Rutelinae	Anomalini	16,805

Bold indicates the species newly sequenced in this study.

## Data Availability

The newly sequenced mitochondrial genomes have been submitted to the NCBI GenBank database, with the accession numbers of PZ322948-PZ322950.

## Supplementary Materials

The following supporting information can be downloaded at: https://www.mdpi.com/article/10.3390/biology15120953/s1, Figure S1: Gene arrangement of the 44 mitogenomes analyzed in this study. Figure S2: Structural organization of the mitogenomic control regions of the three newly sequenced Dynastinae species (*Cylochelus signaticollis*, *Dasygnathus* sp., and *Xastronomes australicus*). Figure S3: ML tree inferred from the 44taxa_PCG_nt123 nucleotide dataset. Numbers at nodes represent bootstrap values. Figure S4: ML tree inferred from the 44taxa_PCG_aa amino acid dataset. Numbers at nodes represent bootstrap values. Figure S5: BI tree inferred from the 44taxa_PCGrRNA nucleotide dataset. Numbers at nodes represent Bayesian posterior probabilities. Figure S6: BI tree inferred from the 44taxa_PCG_nt123 nucleotide dataset. Numbers at nodes represent Bayesian posterior probabilities. Figure S7: BI tree inferred from the 44taxa_PCG_aa amino acid dataset. Numbers at nodes represent Bayesian posterior probabilities. Table S1: Summary of whole genome sequencing statistics for the species analyzed in this study. Table S2: Organization of the newly sequenced mitogenomes. Supplementary Material: Nucleotide sequences of the three newly sequenced mitogenomes (FASTA format).

## Abbreviations

The following abbreviations are used in this manuscript:

ATP	Adenosine triphosphate
Mitogenome	Mitochondrial genome
PCGs	Protein-coding genes
tRNAs	Transfer RNAs
DNA	Deoxyribonucleic acid
BOLD	Barcode of Life Data System
ID%	Identity percentage
Bp	Base pair
RSCU	Relative synonymous codon usage
ML	Maximum likelihood
BI	Bayesian inference
BS	Bootstrap support
BPP	Bayesian posterior probabilities
CR	Control region
