# Supplementary figures and images for: Complete Mitochondrial Genomes of Three Rhinoceros Beetles (Coleoptera: Scarabaeidae: Dynastinae) and Phylogenetic Implications
Source: Biology (Basel). 2026 Jun 18;15(12):953. doi: 10.3390/biology15120953 (PMC13295536; doi:10.3390/biology15120953)

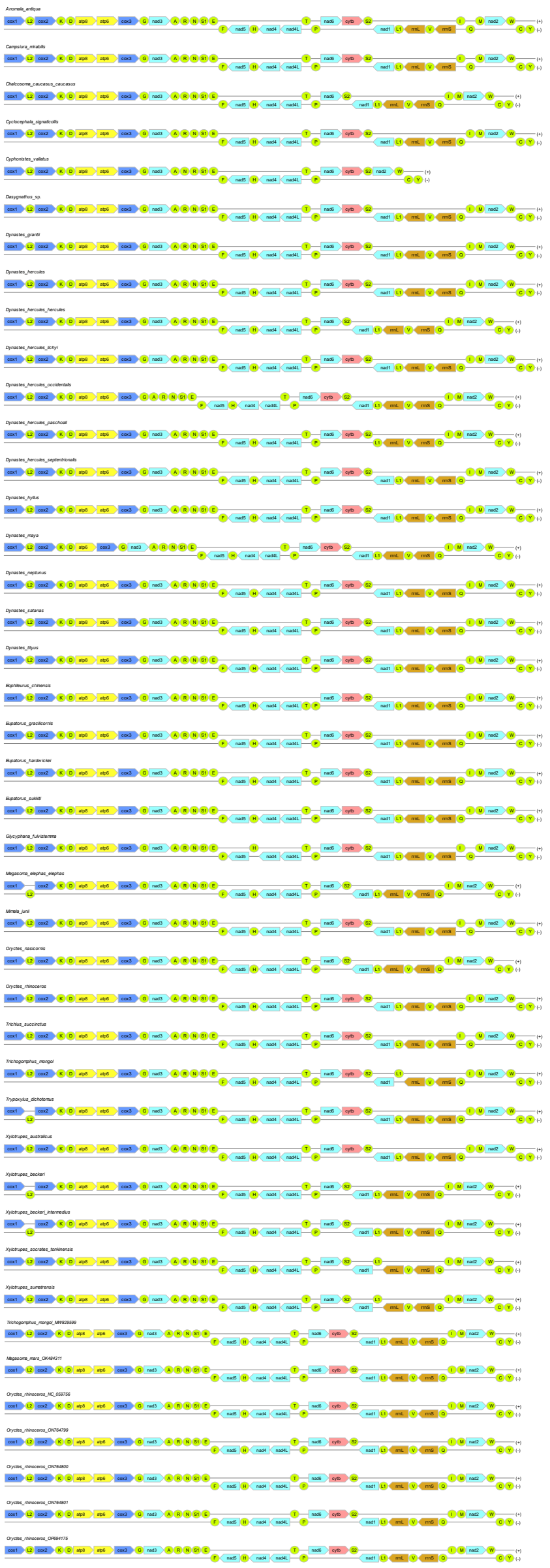

Supplement: Supplementary file 1 [file biology-15-00953-s001.zip › Figure S1.pdf]

1. *Cylochelus signaticollis*

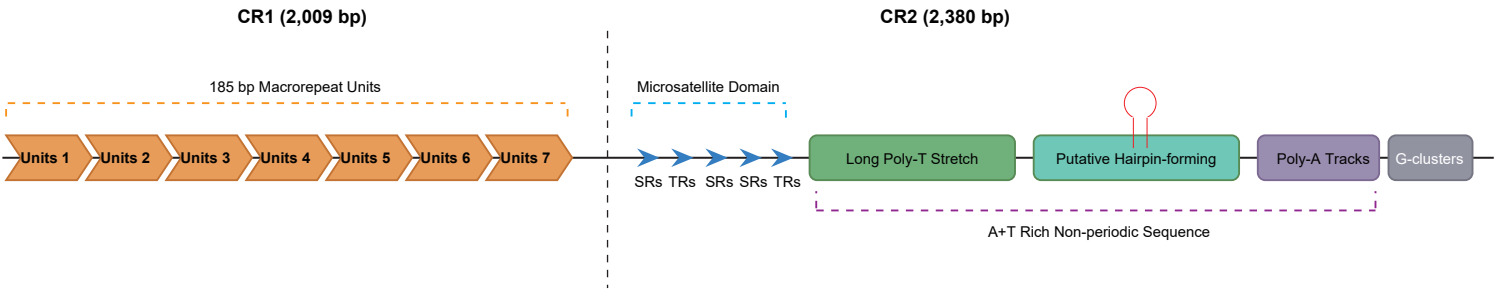

2. *Dasygnathuss* sp.

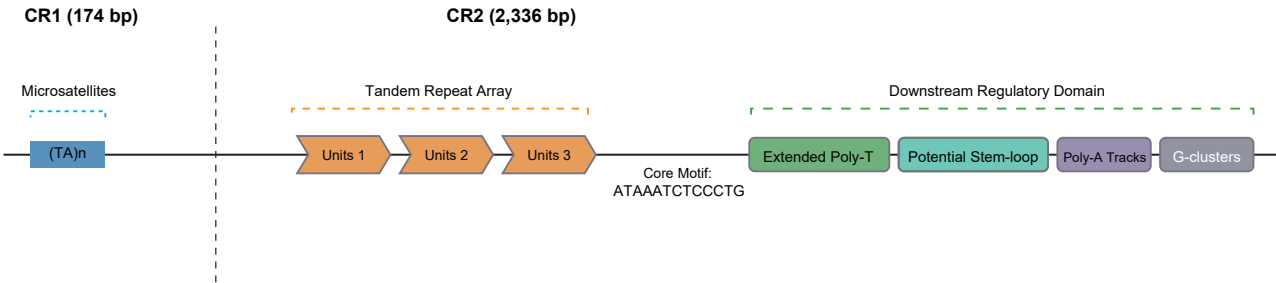

3. *Xastronomes australicus*

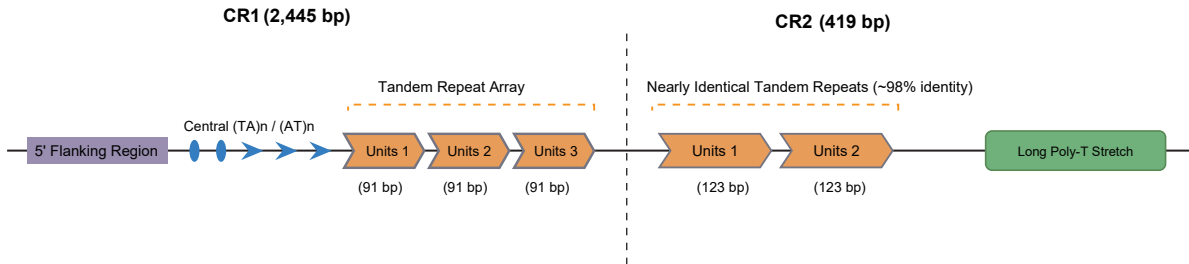

Supplement: Supplementary file 1 [file biology-15-00953-s001.zip › Figure S2.pdf]

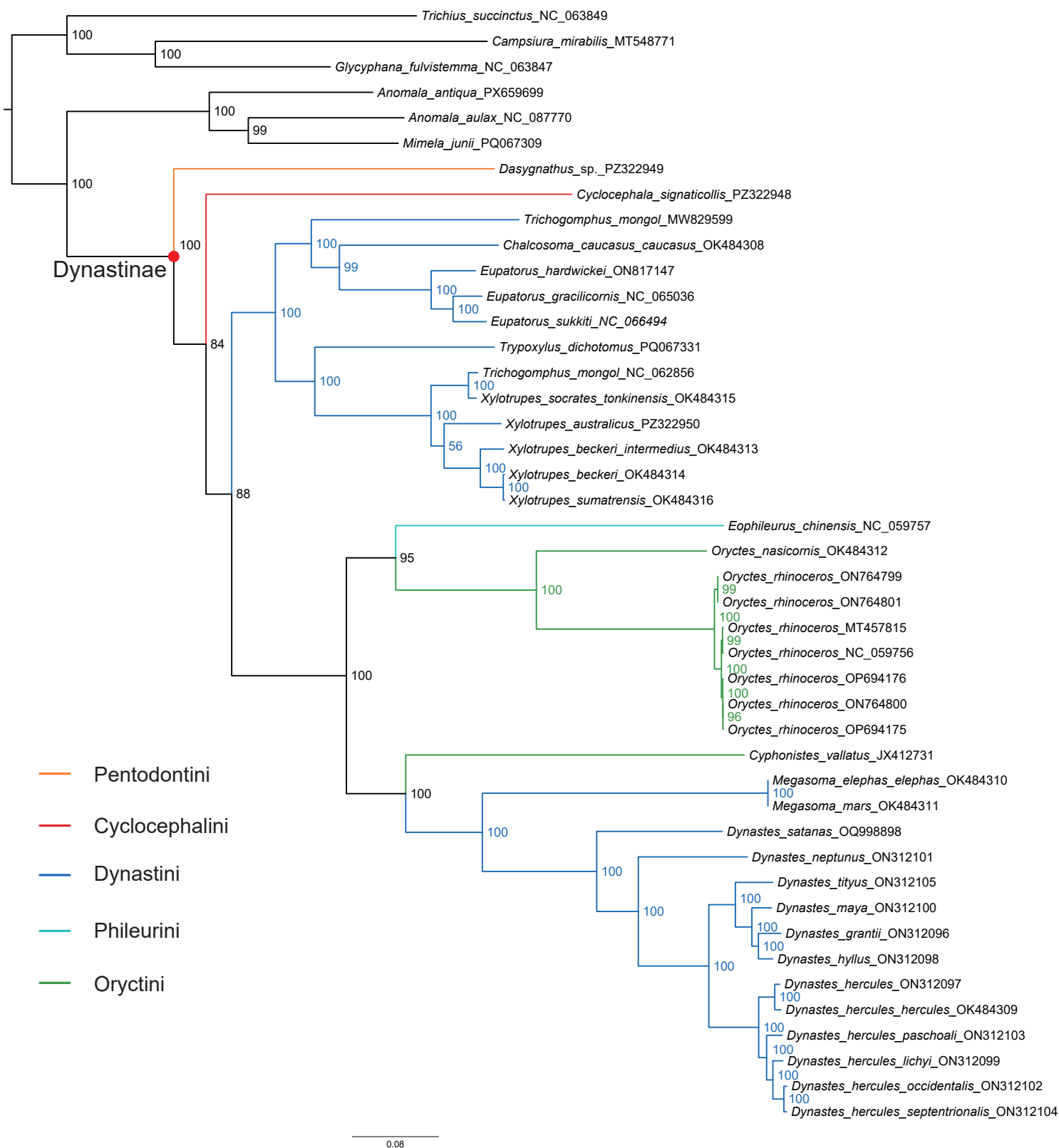

Supplement: Supplementary file 1 [file biology-15-00953-s001.zip › Figure S3.pdf]

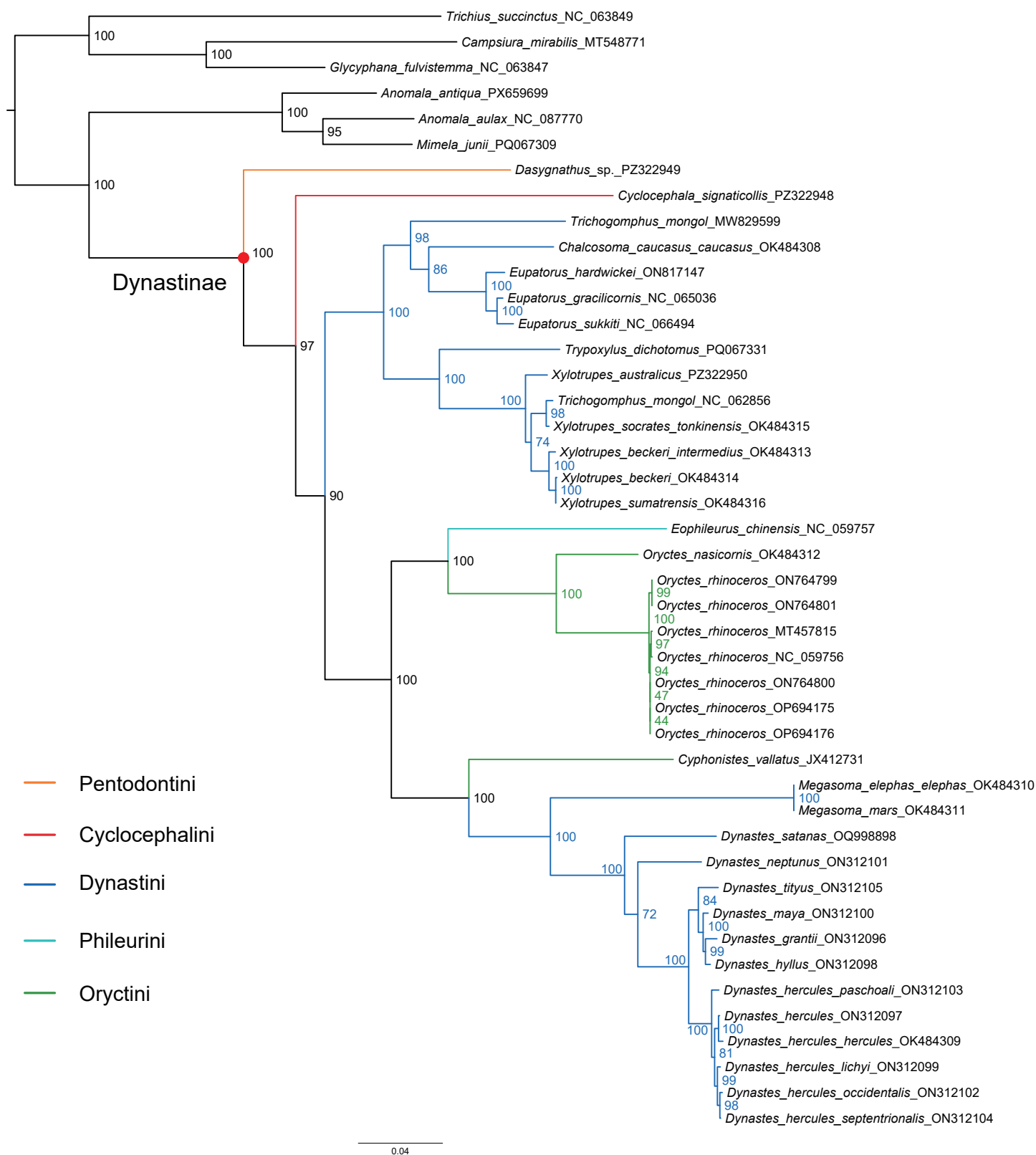

Supplement: Supplementary file 1 [file biology-15-00953-s001.zip › Figure S4.pdf]

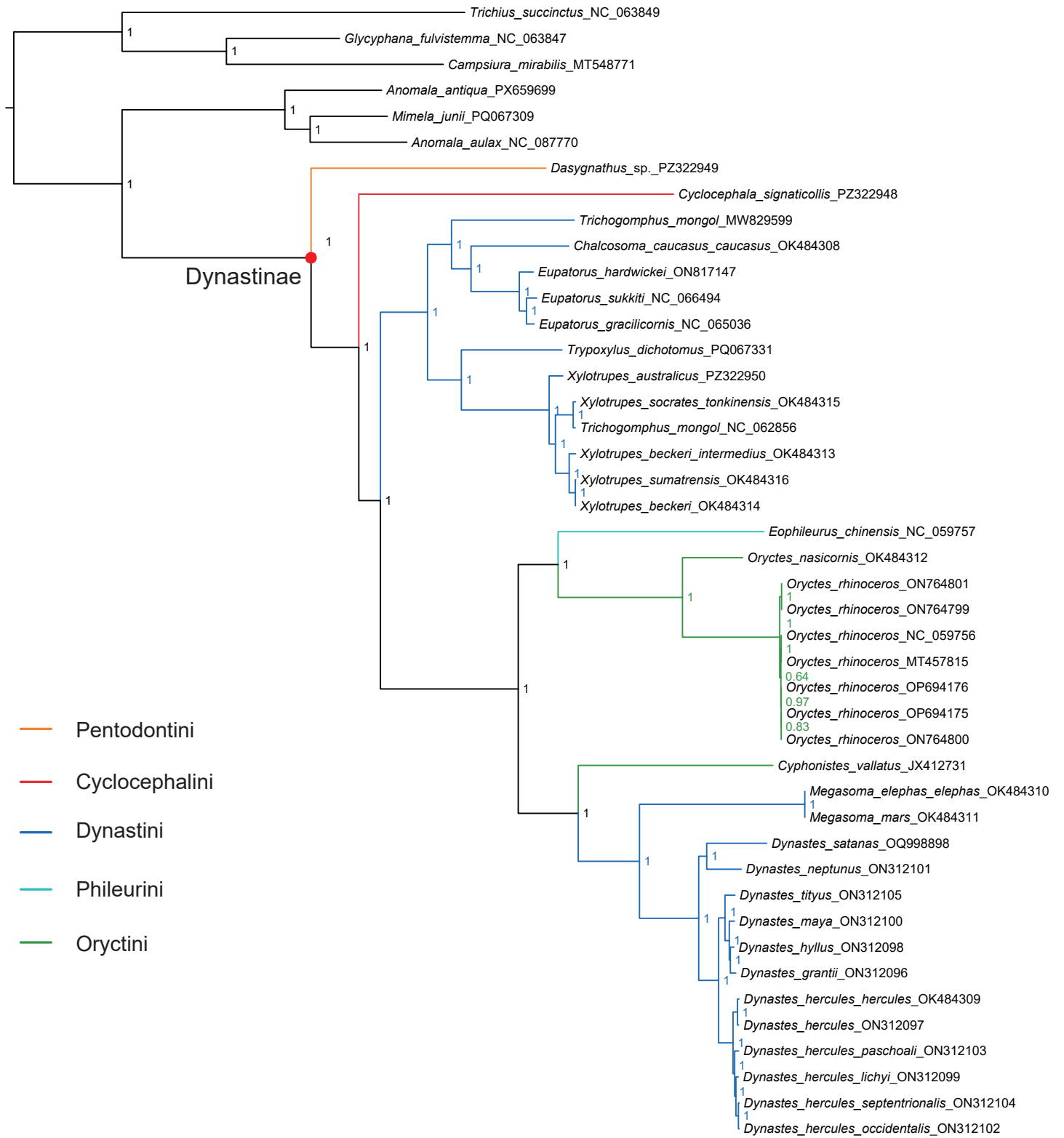

0.3

Supplement: Supplementary file 1 [file biology-15-00953-s001.zip › Figure S5.pdf]

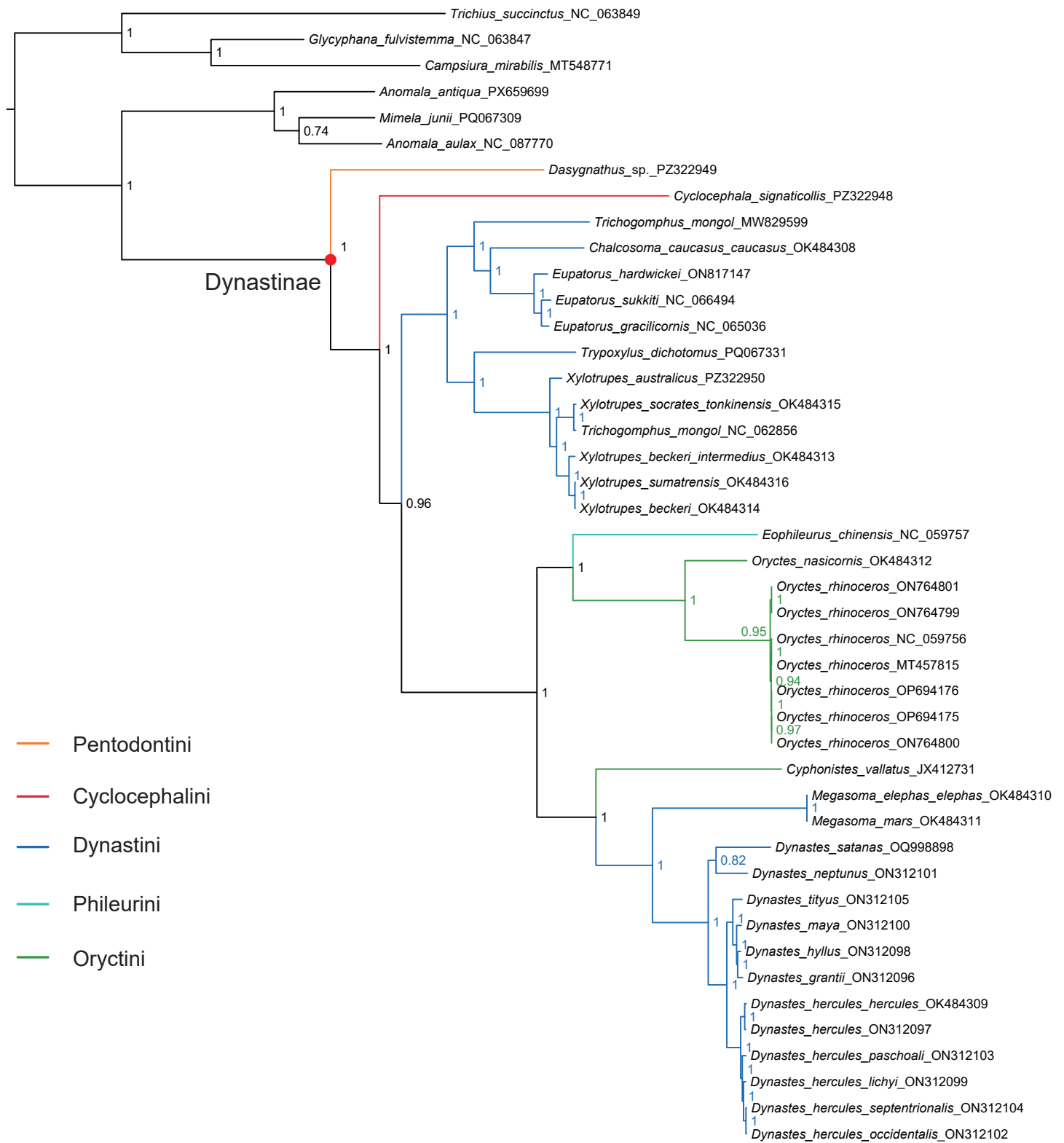

0.4

Supplement: Supplementary file 1 [file biology-15-00953-s001.zip › Figure S6.pdf]

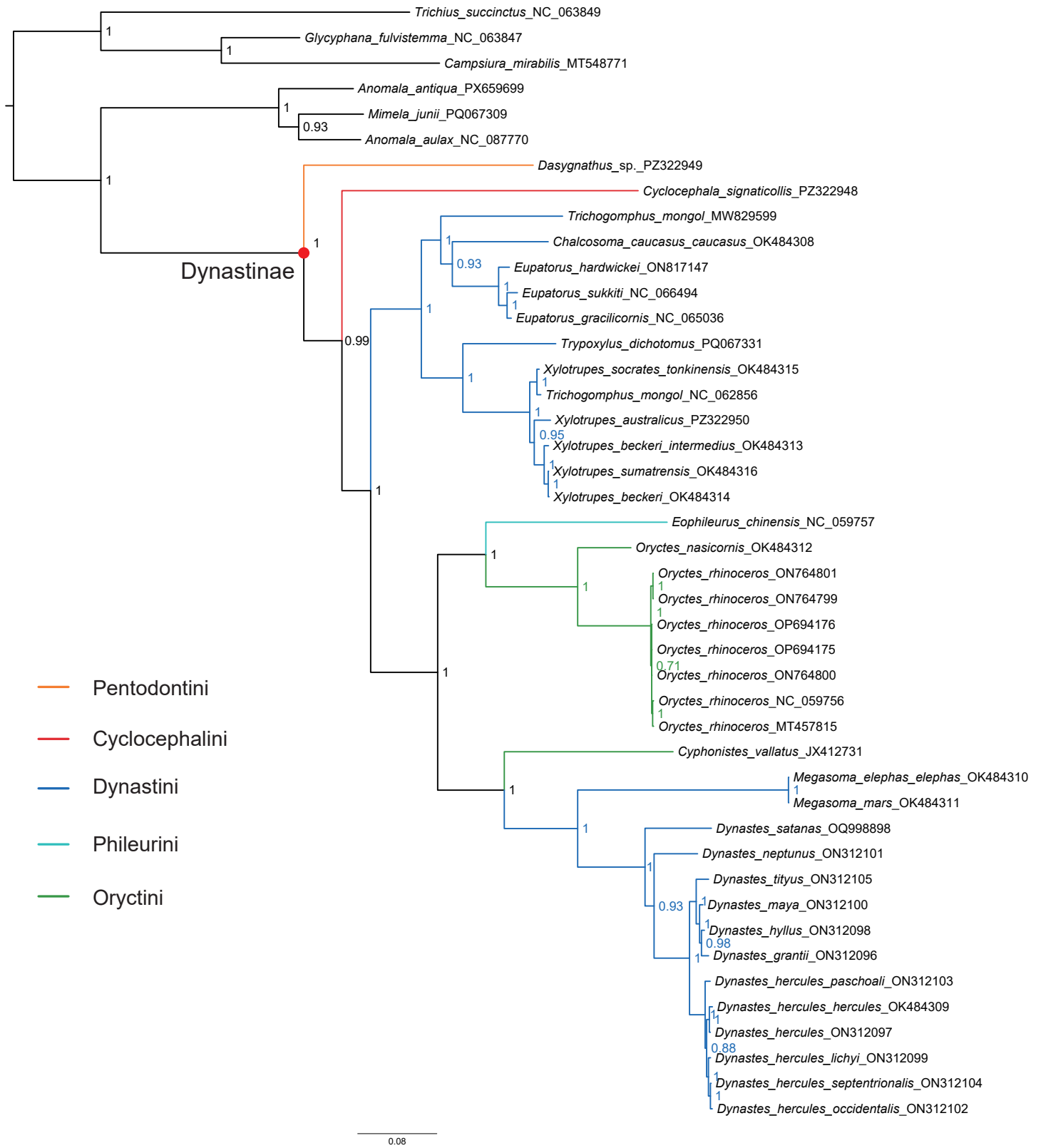

Supplement: Supplementary file 1 [file biology-15-00953-s001.zip › Figure S7.pdf]
